# Hypothalamic atrophy in progressive supranuclear palsy, assessed by convolutional neural network-based automatic segmentation

**DOI:** 10.1007/s00415-026-13718-z

**Published:** 2026-03-11

**Authors:** Jan Kassubek, Günter U. Höglinger, Adam Zůza, Kornelia Kreiser, Francesco Roselli, the DESCRIBE-PSP Study Group, Günter U. Höglinger, Moritz Brandt, Katharina Buerger, Emrah Düzel, Björn Falkenburger, Agnes Flöel, Wenzel Glanz, Daniel Janowitz, Sabrina Katzdobler, Ingo Kilimann, Okka Kimmich, Johannes Levin, Oliver Peters, Josef Priller, Johannes Prudlo, Luisa-Sophie Schneider, Annika Spottke, Eike Jakob Spruth, Matthis Synofzik, Stefan Teipel, Carlo Wilke, Hans-Peter Müller

**Affiliations:** 1 Department of Neurology, University of Ulm, Oberer Eselsberg 45, 89081 Ulm, Germany; 2 Department of Neurology, LMU University Hospital, Ludwig-Maximilians-Universität (LMU), Munich, Germany; 3 German Center for Neurodegenerative Diseases (DZNE), Site Munich, Munich, Germany; 4 Munich Cluster for Systems Neurology (SyNergy), Munich, Germany; 5 SLK-Kliniken Heilbronn GmbH, Heilbronn, Germany; 6 Department of Diagnostic and Interventional Radiology, University Hospital Ulm, Ulm, Germany; 7 German Center for Neurodegenerative Diseases (DZNE), Site Ulm, Ulm, Germany; 8 Department of Nuclear Medicine, University Hospital Ulm, Ulm, Germany

**Keywords:** Hypothalamus, Neural networks, Metabolism, Magnetic resonance imaging, Volumetry, Progressive supranuclear palsy

## Abstract

**Background:**

The hypothalamus as one of the core structures in metabolic control is increasingly recognized to be morphologically altered in various neurodegenerative diseases.

**Objective:**

The purpose of this study was to quantitatively investigate the hypothalamic volumes in patients with progressive supranuclear palsy (PSP) and to compare them with controls and Parkinson disease (PD) patients.

**Methods:**

An automatic hypothalamic volume quantification method based on the use of convolutional neural networks (CNN) of U-Net architecture was applied to the automatic segmentation of the hypothalamus and intracranial volumes (ICV). This CNN-based volumetric analysis was performed in high resolution T1 weighted MRI in two PSP cohorts: cohort A with 78 PSP patients and 63 controls was recorded at 3.0 T at multiple sites; the single site cohort B consisted of 66 PSP patients, 66 PD patients, and 44 controls, recorded at 1.5 T.

**Results:**

In cohort A, significant hypothalamic volume reduction was observed in PSP (774 ± 83 mm^3^) when compared to controls (817 ± 74 mm^3^). In cohort B, this result of significant hypothalamic volume reduction was confirmed in PSP (745 ± 102 mm^3^) when compared to controls (831 ± 81 mm^3^); no significant hypothalamic volume reduction was observed in PD (797 ± 98 mm^3^), in support of previous studies.

**Conclusion:**

The CNN-based hypothalamus volume quantification study demonstrated significantly reduced hypothalamus volumes in PSP patients compared to controls and PD, respectively; future studies will address the metabolic profiles of PSP as potential functional correlates.

**Supplementary Information:**

The online version contains supplementary material available at 10.1007/s00415-026-13718-z.

## Introduction

Alterations in metabolism are increasingly recognized as a functionally important symptom complex in neurodegeneration, since the effects of neurodegenerative syndromes extend beyond motor and cognitive function to involve key physiological processes in many neurodegenerative disorders such as frontotemporal dementia (FTD), amyotrophic lateral sclerosis (ALS), and Alzheimer disease (AD) [1]. Especially in neurodegenerative diseases with frontal lobe involvement like FTD [2], emerging evidence suggests that the disease also affects body functions including changes in eating behavior and metabolism, linked to large-scale neural networks involving key structures including the hypothalamus [2]. In general, physiological changes across all these conditions include neuroendocrine pathways, reward pathways, motor systems and the autonomic nervous system, and central to these changes are potentially complex neural networks involving the hypothalamus, with hypothalamic atrophy shown in behavioral variant FTD [2] and ALS [3–5]. At the histopathological level, the supraoptic (SON) and paraventricular (PVN) nuclei of the hypothalamus (which undergo structural and functional changes over the course of healthy aging) have been shown to be heterogeneously affected by several different neurodegenerative diseases including FTD, ALS, AD, and also movement disorders such as progressive supranuclear palsy (PSP), Parkinson disease (PD), dementia with Lewy bodies, and Huntington disease [6].

Hypothalamic structures have been extensively investigated in large patient samples by specifically targeted neuroimaging/magnetic resonance imaging (MRI) in many of these neurodegenerative conditions [2, 4, 5], demonstrating hypothalamus atrophy especially in diseases with frontal involvement as one core feature like FTD and ALS. However, due to the variable involvement, mirrored in the heterogeneity of the histopathological data, hypothalamic atrophy is not a part of the imaging signature at the group level in other neurodegenerative diseases like PD [7, 8]. PSP is a 4R-tauopathy characterized by subcortical tau inclusions in neurons, astrocytes, and oligodendroglia and is associated with various clinical phenotypes [9–11]. The PSP phenotype includes frontal lobe involvement at the functional [12] and the neuroimaging domain [10, 13]. Previous volumetric MRI studies in PSP demonstrated reduced volumes in the ventral diencephalon region which includes the hypothalamus [14–17], indicating that the PSP-associated morphological changes involve this area. We hypothesized that hypothalamus atrophy might be an element of the neuroimaging signature of PSP as a condition with overlap to FTD and performed a quantification of hypothalamic volumes in volume-rendering MRI of a group of PSP patients vs. healthy controls and vs. PD by a novel automatic approach with the use of convolutional neural networks (CNN) [7].

## Material and methods

### Subject cohorts

The analysis comprised two cohorts of patients and healthy controls: cohort A included 78 patients with PSP and 63 controls from the DESCRIBE and DANCER studies of the German Center for Neurodegenerative Diseases (DZNE) at 12 sites (for the distribution of subjects in the different centers, please refer to Supplementary Table 1). Cohort B included a single-site dataset of 66 PSP patients, 66 PD patients, and 44 healthy controls, recorded at Ulm University. Patients and controls were age- and sex-matched (Table 1). All patients from the two cohorts of PSP patients fulfilled the Movement Disorder Society (MDS) 2017 diagnostic criteria [18] and had a certainty level of probable PSP, and the cohorts only included the most common subtypes Richardson syndrome (PSP–RS) and the Parkinsonian variant of PSP (PSP–P).

**Table 1:** Subjects’ characteristics

		*N* (m/f)	Age/years	Disease duration/years
Cohort A (3.0 T)	PSP–RS patients	55 (24/31)	71.0 ± 7.4 (50.6–86.2)	4.3 ± 2.8 (0.6–14.4)
	PSP–P patients	23 (14/9)	68.2 ± 7.6 (52.0–83.9)	3.8 ± 1.8 (0.5–6.9)
	*p* (*t*-test, PSP–RS vs. PSP–P)	–	n.s.	n.s.
	All PSP patients	78 (38/40)	70.0 ± 7.4 (50.6–86.2)	4.2 ± 2.7 (0.5–14.4)
	Controls	63 (30/33)	68.6 ± 7.7 (51.2–89.4)	–
	*p* (controls vs PSP)		n.s. (*t*-test)	
Cohort B (1.5 T)	PSP–RS patients	46 (24/22)	70.0 ± 9.2 (49.0–84.3)	2.9 ± 1.6 (0.5–6.9)
	PSP–P patients	20 (14/6)	70.9 ± 8.9 (50.2–91.3)	3.4 ± 1.6 (1.1–8.7)
	*p* (*t*-test, PSP–RS vs. PSP–P)	–	n.s.	n.s.
	All PSP patients	66 (38/28)	70.5 ± 9.1 (49.0–91.3)	3.1 ± 1.6 (0.5–8.7)
	Controls	44 (25/19)	68.5 ± 5.3 (57.2–81.9)	–
	PD patients	66 (41/25)	70.4 ± 10.4 (52.0–93.5)	3.6 ± 2.6 (0.8–9.7)
	*p* (controls vs PSP vs PD)	–	n.s. (one-way ANOVA)	n.s. (*t*-test)

Values of continuous variables are given in mean ± standard deviation and range

*PSP* progressive supranuclear palsy, *PSP–P* progressive supranuclear palsy with predominant parkinsonism, *PSP–RS* progressive supranuclear palsy with Richardson’s syndrome, *PSPRS* progressive supranuclear palsy rating scale, *PD* Parkinson disease

The study adhered to the principles of the Declaration of Helsinki, and written informed consent was obtained from all participants. The study was approved by the responsible Ethics Committees: for cohort A according to institutional guidelines of the German Center for Neurodegenerative Diseases with reference 311/14 (“Klinische Register-Studie neurodegenerativer Erkrankungen (DESCRIBE)/Vertiefte Phänotypisierung der Progressiven Supranukleären Parese (DESCRIBE–PSP)”) and for cohort B by the Ethics Committee of Ulm University, Germany (with reference 182/23).

### MRI scanning

The MRI protocol for cohort A (recorded at 3.0 T scanners as part of the multicentric DZNE studies DESCRIBE and DANCER) included high-resolution T1-w scans with 192 sagittal slices (slice thickness 1.0 mm, in-plane pixel size 1.0 × 1.0 mm^2^ in a 256 × 256 matrix); echo time (TE) was 4.3 ms and repetition time (TR) was 2500 ms. The MRI protocols had been harmonized across research centers and vendors. The MRI protocol for cohort B (recorded at 1.5 T; Magnetom Symphony, Siemens Healthineers, Erlangen, Germany) included high-resolution T1-w scans with 144 sagittal slices (slice thickness 1.2 mm, in-plane pixel size 1.0 × 1.0 mm^2^ in a 256 × 256 matrix; TE, 4.2 ms, TR, 1640 ms). Only MRI scans without artifacts and without any imaging abnormalities which might compromise the accurate assessment of the scans (like extended vascular lesions) were used for the study.

### Data analysis

#### Pre-processing

The MRI data were pre-processed using the *Tensor Imaging and Fiber Tracking* (TIFT) software package expanded by a volumetric extension package (Version 2025 [19]): a correction for individual tilt of the head in order to minimize potential partial volume effects was performed by a rigid body normalization of T1-w data along the anterior commissure (AC)—posterior commissure (PC) axis with coronal planes perpendicular to the AC–PC axis. To improve the accuracy in identifying hypothalamic borders in the coronal planes, a spatial upsampling was applied to the 50 slices of the hypothalamic Sect. (0.5 mm thickness; matrix 512 × 512, resolution 0.125 × 0.125 mm^2^).

#### Post-processing

The T1-weighted data (1.5 T) used in the current study show basically a similar or identical morphology compared to the data of a previously published deep learning-based pipeline [7]. This pipeline was used for the automatic segmentation of the hypothalamus and the intracranial volume (ICV) which employs convolutional neural networks (CNN) with a U-Net architecture for both structures. CNNs are able to learn hierarchical features representing different levels of abstraction in a data-driven manner. The applicability of this pipeline to T1-weighted data from a 3.0 T scanner is conceptually possible (irrespective of the pathology analyzed), as the 3.0 T data of the current study have a similar resolution with (as an intrinsic property of 3.0 T data) a better signal-to-noise ratio. ICV was used to account for inter-individual differences in head size by normalizing hypothalamic volumes [3]:

$$ H_{\mathrm{norm}} = H/{\mathrm{ICV}}\times 1564\; {\mathrm{mm}}^{3}, $$

where 1564 mm^3^ is the averaged ICV of all subjects contributing to this study.

### Statistical analysis

A quality check of the automated segmentation was performed based on the outliers detected in the ICV-normalized hypothalamus volume. The outliers were identified by applying interquartile range (IQR): any point outside MEDIAN ± 1.5*IQR was considered to be an outlier.

Statistical analysis was performed using Python (v3.11) with statsmodels and scipy libraries. Group differences in hypothalamic volume (ICV normalized) were assessed using Analysis of Covariance (ANCOVA) with age and sex as covariates. Analyses were conducted separately for each cohort (cohort A: 3.0 T MRI; cohort B: 1.5 T MRI). For cohort B, pairwise comparisons between groups (PSP, PD, controls) were performed using separate ANCOVA models for each pair, controlling for age and sex. Statistical significance was set at *p* < 0.05.

Association analysis of normalized hypothalamic volume and disease duration was performed by Pearson correlation.

## Results

In cohort A, 71 out of the 78 data of PSP patients and all data of controls were used for statistical analysis, i.e., 7 data of PSP patients were identified as outliers. In cohort B, 59 out of the 66 data of PSP patients and 58 out of the 66 data of PD patients were used for statistical analysis, i.e., 7 data of PSP patients and 8 data of PD patients were identified as outliers; one of the controls’ data was identified as outlier.

In cohort A, ANCOVA revealed a significant effect of group on hypothalamic volume after controlling for age and sex (*F*(1,130) = 9.17, *p* = 0.003). PSP patients showed significantly reduced hypothalamic volume (mean ± SD, 774 ± 74 mm^3^) compared to controls (817 ± 83 mm^3^). Neither age (*p* = 0.440) nor sex (*p* = 0.256) were significant covariates.

**Fig. 1:**
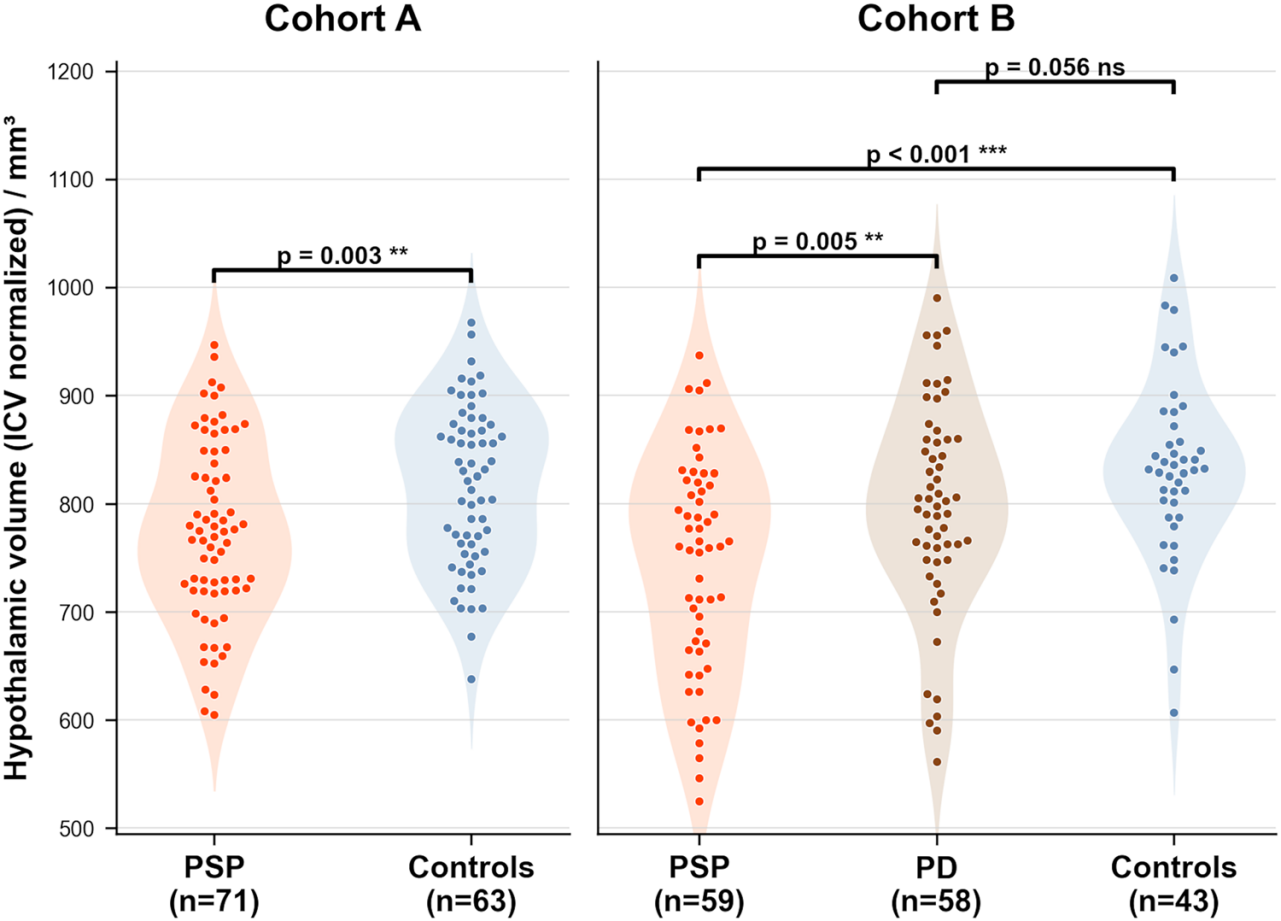
ICV-normalized hypothalamic volumes at the group level for cohorts A and B. Group differences were assessed using ANCOVA controlling for age and sex. Cohort A: PSP vs controls, *p* = 0.003. Cohort B: PSP vs controls, *p* < 0.001; PSP vs PD, *p* = 0.005; PD vs controls, *p* = 0.056 (not significant (n.s.)). *PSP* progressive supranuclear palsy, *PD* Parkinson disease. **p < 0.01, ***p < 0.001

In cohort B, ANCOVA revealed a significant overall effect of group (*F*(2,155) = 11.44, *p* < 0.001). Pairwise comparisons showed that PSP patients had significantly lower hypothalamic volumes (745 ± 102 mm^3^) than both controls (831 ± 81 mm^3^) and PD patients (797 ± 98 mm^3^), respectively. The difference between PD patients and controls did not reach statistical significance (*p* = 0.056). Sex was a significant covariate in cohort B (*F*(1,155) = 5.45, *p* = 0.021), whereas age was not with *p* = 0.804. These results (Fig. 1) were in support of the results of previous studies [7, 8]. These findings demonstrate consistent hypothalamic volume reduction in PSP patients across two independent cohorts, independent of age and sex effects.

No association could be found for normalized hypothalamic volume and disease duration.

## Discussion

The unbiased CNN-based analysis of the hypothalamus volumes in PSP demonstrated a significant hypothalamic atrophy, in comparison both to healthy controls and a disease control group of PD patients. In line with the hypothesis, these results are in support of the hypothalamic involvement of PSP as a neurodegenerative disease, especially as a condition with frontal symptoms and overlap to the FTD spectrum diseases [12]. In contrast to the FTD spectrum entities, the neurodegenerative process of PD as a condition with less prominent frontal involvement is not associated with a significant hypothalamic atrophy at group level, in line with the limited and heterogeneous neuropathological data [6].

At the structural level, the hypothalamus is part of the ventral diencephalon which was demonstrated to be altered in PSP in previous advanced MRI studies [14–17] so that hypothalamic alterations can be considered to be part of the neuroimaging signatures of PSP. Given that the ventral diencephalon extends beyond the hypothalamus, a volumetric analysis specifically targeted at the hypothalamus structure like the current one can quantify its structural alterations, all the more since its low size and anatomical localization prevents it to be detected in many volumetric MRI measurements at whole brain level [20]. The applicability of this pipeline to T1-weighted data from a 3.0 T scanner showed, probably due to the better signal-to-noise ratio, less outliers compared to the 1.5 T data. The results of the current study are in accordance with the (scarce) histopathological data addressing specifically the hypothalamus in PSP, i.e., the finding of disease-specific pathological inclusions in the suprachiasmatic nucleus (SCN) of the hypothalamus [21] in a group of PSP patients.

Although hypothalamic atrophy in PSP could implicate associations with disease duration, no association could be found for normalized hypothalamic volume and disease duration. Regarding reasons for this lack of association, it is of note that disease duration is a blurry variable in progressive neurodegenerative diseases per se and that the small distribution range of values for disease duration in the given samples (3.6 ± 2.1 years) might further reduce the validity of this association analysis. Our data, thus, do not allow statements about a possible temporal relationship of hypothalamic atrophy in the progressive cerebral volume changes during the disease course of PSP.

The major further conclusions beyond structural volume loss have to be drawn at the functional level, especially with respect to metabolism; however, these data are lacking in the current neuroimaging-based study (see limitations paragraph). Metabolic alterations have been demonstrated in PSP, i.e., in a pilot study in 15 PSP patients, the authors presented reduced caloric and proteins intake regardless the presence of dysphagia [22]. Future longitudinal studies on body composition should be performed in PSP, although weight loss is a less prominent feature in PSP e.g. compared with other diseases with early hypothalamic involvement like ALS. Then, correlational analyses with hypothalamic atrophy might be added, together with hypothalamic functional assessments. In that context, oxytocin might be addressed which is produced in the hypothalamus and which has been shown to be altered in the pathology of HD, ALS, and FTD (as neurodegenerative conditions with hypothalamus involvement) [23]. No specific studies on hypothalamic functions were reported in PSP yet, but such data might be used to identify commonalities across various neurodegenerative conditions, perhaps with the potential option of an integration into therapeutic concepts in PSP.

The strengths of the current study include the dedicated unbiased, quantitative measurements of the hypothalamic volumes in two sizeable groups of PSP patients in a design with a study cohort (cohort A) and a validation cohort (cohort B) by use of a deep learning model (CNN) for the analysis. Furthermore, in preparation targeting to automated clinical application a strict outlier detection was used without any manual post-analysis corrections. The major limitation is the lack of correlations with hypothalamic functional assessments such as metabolism (body weight/body mass index and body composition), eating behavior, and sleep. Prospective, especially longitudinal data are needed in future studies to explore what PSP-associated hypothalamic atrophy might mean in the functional domain.

In summary, this systematic deep learning-based volumetric quantification of hypothalamic atrophy demonstrated that volume loss of the hypothalamus is an element of the pattern of neuropathological alterations associated with PSP. This atrophy of the hypothalamus is a common element with other neurodegenerative conditions including those with frontal involvement (like FTD or ALS) or other neurodegenerative diseases, but differs from others like PD (as confirmed in the current study). The consequences of the hypothalamus involvement for a deeper understanding of PSP pathophysiology on the one hand and, at the clinical level, for patient care with respect to hypothalamically controlled functions like metabolism or sleep on the other hand have to await future studies.

## Electronic supplementary material

Below is the link to the electronic supplementary material.

Supplementary file1 (PDF 45 KB)

## Data Availability

Reasonable data sharing requests can be addressed to the corresponding author and require a formal data sharing agreement with the DZNE and the University Hospital Ulm.
