# Supplementary material for: Hypothalamic atrophy in progressive supranuclear palsy, assessed by convolutional neural network-based automatic segmentation
Source: J Neurol. 2026 Mar 11;273(3):201. doi: 10.1007/s00415-026-13718-z (PMC12979338; doi:10.1007/s00415-026-13718-z)
Supplement: Supplementary file 1 — Supplementary file1 (PDF 45 KB) [file 415_2026_13718_MOESM1_ESM.pdf]

Supplementary Table 1: In cohort A, data were collected from 12 different centers: models of the MR tomographs and participants' distribution at the respective center.

**center no.**	**model**	**controls**	**PSP**
1	Siemens TrioTim	16	10
2	Siemens Verio	10	2
3	Siemens TrioTim	31	
4	Siemens Skyra	3	
5	Siemens Biograph_mMR	3	
6	Siemens Skyra fit		29
7	Siemens TrioTim		24
8	Siemens Skyra		6
9	Siemens Verio		4
10	Siemens Prisma fit		1
11	Siemens Verio		1
12	Siemens Verio		1
**Sum**		**63**	**78**
